# Advances and challenges in avian influenza vaccines: From conventional approaches to next-generation strategies

**DOI:** 10.1016/j.vas.2026.100883

**Published:** 2026-09-27

**Authors:** Mahboobeh Nazari, Mohammad Mahdi Mehrazar

**Affiliations:** Monoclonal Antibody Research Centre, Avicenna Research Institute, ACECR, Tehran, Iran

**Keywords:** Avian influenza virus, Aiv vaccines, Highly pathogenic avian influenza, DIVA, Mucosal immunity, Antigenic drift, Poultry vaccination

## Abstract

**Background:**

Avian influenza viruses (AIVs), particularly highly pathogenic H5 and H7 viruses, pose major threats to poultry production, food security, international trade, and public health. Vaccination is an important component of AIV control; however, antigenic evolution, incomplete reduction of viral shedding, and challenges associated with large-scale poultry vaccination limit its effectiveness. In addition to conventional vaccines, recombinant and nucleic-acid platforms have emerged in response to rapidly evolving AIVs.

**Methods:**

Relevant literature on inactivated, live attenuated, recombinant subunit, viral-vector, DNA, mRNA, and virus-like particle vaccines was evaluated. Immune responses, protection, antigenic breadth, viral shedding, delivery, manufacturing, field applicability, and regulatory considerations were analyzed.

**Results:**

Inactivated vaccines are well-established, safe, scalable, and cost-effective but provide limited cellular and mucosal immunity and require antigenic matching. Live attenuated vaccines can induce broader immunity and facilitate mass vaccination but present biosafety, reassortment, and genetic-stability concerns. Next-generation platforms provide greater antigenic flexibility and opportunities for broader protection. Viral-vector vaccines can induce both humoral and cellular immunity, while recombinant subunit, DNA, mRNA, and VLP vaccines offer rapid redesign and multivalent formulations. However, challenges remain regarding delivery, stability, manufacturing, durability, and field validation.

**Conclusions:**

No single platform currently meets all requirements for sustainable AIV control. Future strategies should emphasize conserved or mosaic antigens, durable and mucosal immunity, rapid vaccine updating, DIVA compatibility, and scalable delivery. Combining conventional and emerging platforms through prime-boost or multi-platform strategies may provide more flexible and effective control.

**Abbreviations**
AIV: avian influenza viruses
BEVS: baculovirus expression vector system
bNAbs: Broadly-Neutralizing Antibodies
CpG ODN: CpG oligodeoxynucleotides
CTLs: cytotoxic T lymphocytes
DGL: dendritic polylysine
DIVA: Differentiating Infected from Vaccinated Animals
dpc: days post challenge
FliC: flagellin
Gs/GD: goose/Guangdong
GM-CSF: granulocyte-macrophage colony-stimulating factor
HI: hemagglutination inhibition
HA: hemagglutinin
HVT: herpesvirus of turkey
HPAI: highly pathogenic avian influenza
IFN-γ: interferon gamma
LNPs: lipid nanoparticles
LAIVs: Live attenuated avian influenza vaccines
LPVI: low-pathogenic avian influenza
mH7: membrane-bound HA
MDCK: Madin-Darby canine kidney
MREs: microRNA response elements
MPLA: monophosphoryl lipid A
MLV: mouse leukemia virus
MBS: multi-basic cleavage site
NKT: natural killer T
NA: neuraminidase
bnAbs: neutralizing antibodies
bnAbs: neutralizing antibodies
NS1: nonstructural protein 1
RBS: receptor-binding sites
RBS: receptor-binding sites
sa-mRNA: Self-amplifying mRNA
TS: temperature-sensitive
FAO: the Food and Agriculture Organization
WOAH: the World Organisation for Animal Health
VLPs: Virus-Like Particles
WHO: World Health Organization
α-GalCer: α-galactosylceramide

## 1 Introduction

### 1.1 Role of vaccination in control and eradication strategies

Vaccination is an effective measure in Avian influenza virus (AIV) control as it not only helps to minimize clinical disease, but also the shedding of the virus and transmission among poultry populations (Lee & Suarez, 2005; Capua & Cattoli, 2007; Marangon et al., 2008). It may be used as a routine and emergency vaccination during outbreaks or preventive vaccination when there is a high risk of virus introduction (Capua & Cattoli, 2007; Marangon et al., 2008).

Vaccination should be combined with other control measures however, such as biosecurity, quarantine, surveillance, education, and prompt killing of infected and/or high-risk poultry (Capua & Cattoli, 2007; Marangon et al., 2008; Hasan et al., 2016). Differentiating Infected from Vaccinated Animals (DIVA) is especially relevant for surveillance and disease control in vaccinated animals (van Oirschot, 1999; Capua et al., 2004). It is possible to pair DIVA-compatible vaccines with companion diagnostic tests to help differentiate infected from vaccinated birds and aid in identification and management of infected flocks (Capua & Cattoli, 2007; Hasan et al., 2016; Śmietanka & Minta, 2010). Good vaccination and eradication programmes also depend on national and regional level legislation, surveillance and rapid-response mechanisms (Bruschke et al., 2007; Lubroth, 2007; Marangon et al., 2007).

The ability of AIV to continuously evolve is a significant factor that makes vaccines less effective. Vaccine formulations must therefore be periodically changed to ensure protection against the current circulating strains, and research is therefore underway to develop vaccines with greater and longer protection (Lee & Suarez, 2005; Kang et al., 2011; Gamoh & Nakamura, 2015; He et al., 2016). Logistical, financial and human resources are also a major constraint in implementing vaccination programmes, and evidence-based policy and vaccinology are critical for successful implementation (Marangon et al., 2008; Lubroth, 2007; Baradaran et al., 2019). Furthermore, vaccination may make surveillance more difficult and interfere with the trade of poultry if traditional diagnostic tests are unable to differentiate vaccinated birds from infected birds. As a result, the vaccination policies are typically established with certain circumstances that take into account disease control, surveillance, and trade conditions (Hasan et al., 2016; Peyre et al., 2009) (supplementary figure 1).

The need for one health is apparent to enhance preparedness to zoonotic spillover and to help maintain sustainable AIV control, by incorporating animal, human and environmental health.

### 1.2 Global burden of avian influenza (H5, H7, H9, other subtypes)

AIVs are a threat to animal and public health, especially the H5, H7 and H9 subtypes, globally. These outbreaks have had significant economic impacts, affected poultry production, and raised the likelihood of zoonotic transmission, in both domestic poultry and wild birds. H5 and H7 viruses are usually associated with highly pathogenic avian influenza (HPAI) and H9 viruses are classified as low-pathogenic avian influenza viruses (LPAIs), but can still lead to significant production losses and secondary infections. Wild aquatic birds are the natural hosts of AIVs and are important for virus evolution, reassortment and long-distance spread via migratory flyways (Rehman et al., 2023; Shi et al., 2023; Zhirnov & Lvov, 2024).

Of all AIVs, H5 viruses, especially H5N1 and H5N8, are the most significant due to their wide geographical distribution, high pathogenicity and zoonotic potential (Fig. 1; Supplementary Table 1). A contemporary H5 HPAI lineage began with the isolation of A/goose/Guangdong/1/1996 (Gs/GD) from a goose in the southern region of China in 1996 (Xu et al., 1999). The hemagglutinin (HA) gene has since seen a great deal of evolution, generating many genetic clades. The World Health Organization (WHO), the World Organization for Animal Health (WOAH), and the Food and Agriculture Organization (FAO) developed a system of phylogeny-based classification of Gs/GD derived H5 viruses to standardize nomenclature and make easy global monitoring (Fig. 1A) (Toward a unified nomenclature system for highly pathogenic avian influenza virus (H5N1), 2008).

**Fig. 1:**
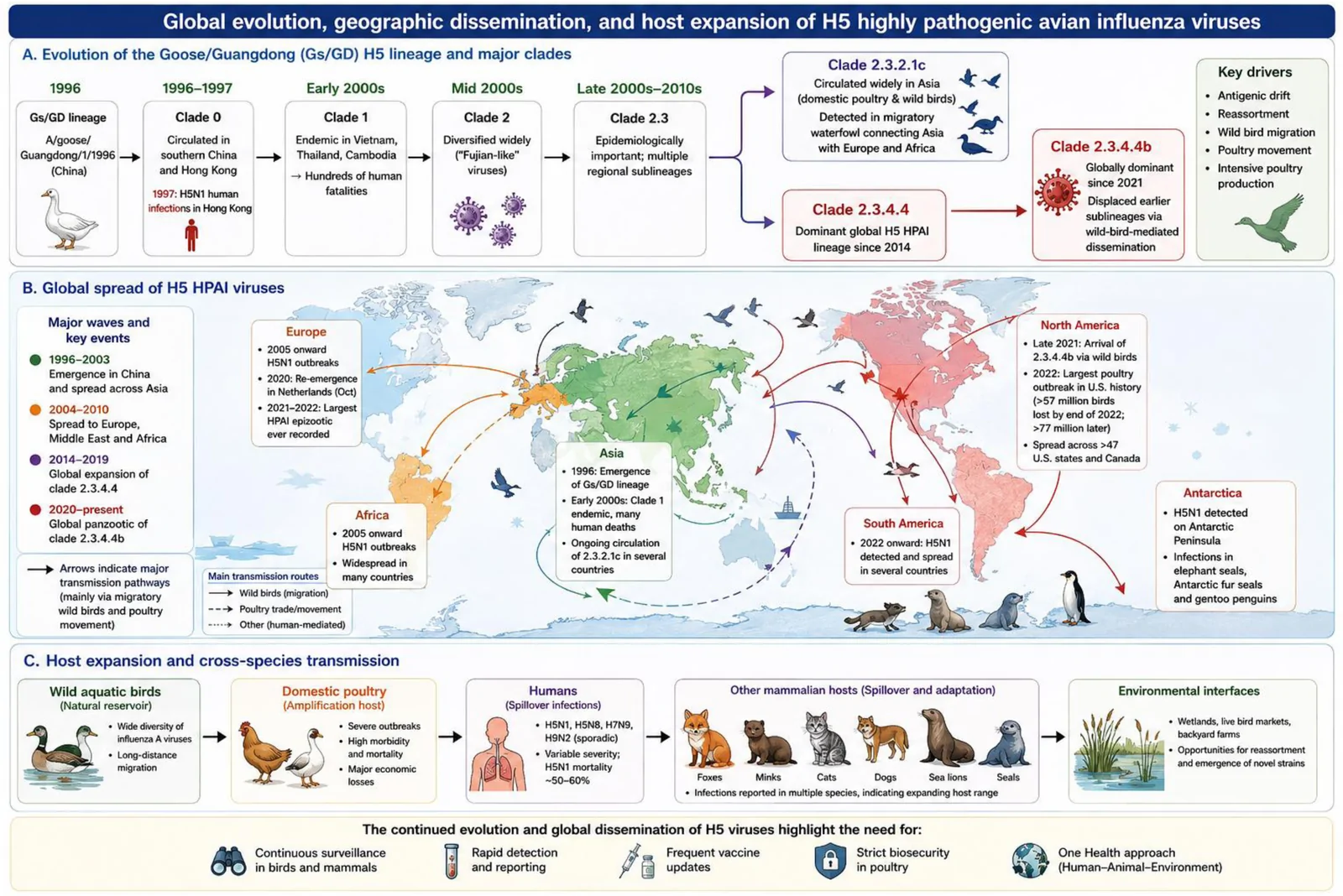
Global evolution, geographic dissemination, and host expansion of H5 highly pathogenic avian influenza (HPAI) viruses. The figure illustrates the evolutionary history of the Goose/Guangdong (Gs/GD) H5 lineage since its emergence in China in 1996. The lineage initially diversified into clades 0, 1, and 2, with subsequent evolution producing major subclades including 2.3.2.1c and 2.3.4.4b. Clade 2.3.2.1c circulated predominantly in Asia, whereas clade 2.3.4.4b emerged as the globally dominant lineage following its expansion after 2020. Wild migratory birds facilitated long-distance transmission across Asia, Europe, Africa, North America, South America, and Antarctica, resulting in the largest recorded HPAI panzootic. The figure also highlights cross-species transmission from wild birds to domestic poultry, humans, and other mammalian hosts, including foxes, minks, cats, sea lions, elephant seals, and Antarctic fur seals. The continued evolution and global dissemination of H5 viruses emphasize the importance of coordinated surveillance, rapid detection, vaccine strain updates, and a One Health approach for effective prevention and control.

The original Gs/GD virus evolved into clade 0, which caused the first documented human H5N1 infections in Hong Kong in 1997 (Chan, 2002; Sims et al., 2005; Xie et al., 2023). Continued evolution generated clade 1 viruses that became endemic in Southeast Asia, followed by clade 2 and several epidemiologically important descendants, including clade 2.3.2.1c and clade 2.3.4.4 (Lee et al., 2017).

Since 2014, clade 2.3.4.4 has become the dominant global H5 lineage, while clade 2.3.4.4b has predominated since 2021 because of its efficient spread by migratory birds across Eurasia, Africa, and the Americas (Xie et al., 2023; Kandeil et al., 2023). Re-emergence of this lineage in Europe during 2020 triggered the largest recorded HPAI epizootic, followed by rapid dissemination throughout North America, South America, and Antarctica, with infections reported not only in poultry and wild birds but also in several mammalian species, including elephant seals and Antarctic fur seals (Kandeil et al., 2023; Green et al., 2023; Alexakis et al., 2024; Ukita et al., 2025). Although clade 2.3.2.1c continues to circulate in parts of Asia, it is increasingly displaced by clade 2.3.4.4b because of its greater ecological fitness (Fig. 1C and Supplementary Table 2) (Tian et al., 2023; Furey et al., 2024).

H5N8 has also emerged as an important HPAI subtype, accounting for >80% of reported global outbreaks in 2020 and spreading to nearly 50 countries (Tsukamoto, 2012; Mekkawy et al., 2025; Purcell et al., 2025). The first confirmed human H5N8 infection, reported in Russia in 2021, further emphasized its zoonotic potential (Ding et al., 2022). H5 outbreaks generally peak between November and May, coinciding with seasonal migration of wild birds. (Host-based classification of influenza viruses is summarized in Supplementary Table 2, while the global evolution and dissemination of H5 HPAI viruses are illustrated in Fig. 1 and Supplementary Table 1).

The H7 lineage includes genetically diverse viruses, such as H7 (N1, N2, N3, N4, N7, and N9), that circulate in wild birds, poultry, and occasionally mammals (Belser et al., 2009; Naguib et al., 2019). These viruses readily undergo mutation and reassortment, allowing low-pathogenic strains to evolve into highly pathogenic variants. Among them, H7N9 has posed the greatest public health concern since its emergence in humans in 2013, causing numerous severe infections, whereas other H7 subtypes have been associated with localized poultry outbreaks and sporadic human infections (supplementary Fig. 2) (Belser et al., 2009).

H9N2 is the most widespread LPAIV subtype worldwide and has considerable veterinary and public health importance despite its relatively low pathogenicity. First identified in turkeys in the United States in 1966, H9N2 subsequently became established in poultry throughout Asia and the Middle East after adapting from aquatic birds (Arbani et al., 2023). H9N2 viruses can be phylogenetically divided into two lineages, North American and Eurasian, with the latter consisting of G1-like, Y280-like and Korean-like groups. G1-like viruses are widely distributed in East Asia, Central Asia and Middle East including Iran, while Y280-like viruses are mainly distributed in China and Southeast Asia. Continuous viral evolution has resulted in frequent emergence of new genetic variants (Guan et al., 1999; Aamir et al., 2007). Evolutionary history, genetic diversity, and global distribution of H9 and H7 avian influenza virus lineages are summarized in (supplementary Fig. 2).

### 1.3 Economic and public health implications (poultry industry losses, zoonotic potential, pandemic risk)

The impact of an outbreak of AIV on poultry production, trade, food security and zoonotic disease risk is significant. Direct losses are the result of infected/exposed birds being culled, production losses, and disease-control measures, while indirect losses are from restrictions on trade, production disruption and consumer confidence losses. For instance, during the 2014–2015 H5Nx outbreak in the USA, the total economic losses were estimated to be $3.3 billion of which the cost to the government amounted to around $850 million (Swayne et al., 2017). Likewise, there were HPAI outbreaks in 2022–2023 in Japan, causing the death of 17.7 million birds and with total economic losses estimated at around 16.9 billion yen (USD 126 million) (Ukita et al., 2025).

The economic impact could be even greater in developing countries, where poultry is important to household economy and nutrition. Fewer than 5% of consumers continued to buy poultry after the Nigerian outbreak in 2006, resulting in significant economic difficulties (Oyekale & Shittu, 2012). The 2006–2007 outbreaks in Ghana led to 51% decrease in consumption of chicken meat and 64% decrease in egg consumption which cost Ghanaians $26.3 million (Subedi et al., 2024; Akunzule et al., 2009). The poultry farmers' income dropped significantly and their profit margins were negative due to similar outbreaks in Turkey, especially among the large-scale farms.

These losses can be further exacerbated by trade restrictions. In the 2014–2015 U.S. HPAI outbreak, bans on U.S. poultry imports were placed by seventeen trading partners with a significant impact on revenue and foreign exchange earnings (Swayne et al., 2017). This is a significant issue, particularly for lower income countries which depend on the production of poultry for food and income (Subedi et al., 2024). In Nigeria, outbreaks of AIV have reduced chicken production by 21 percent and cost farmers an estimated $250 million in lost revenues. Governments also face large costs of disease control and compensation. For example, during the 2006–2007 outbreaks, Ghana experienced approximately $160, 000 in costs for compensation and $4.3 million in disease-control costs (Akunzule et al., 2009).

In addition to economic effects, AIV is a serious public health problem due to its zoonotic potential. Humans may be susceptible to H5N1, and infected humans have reported 50% or more mortality from H5N1 strains that are highly pathogenic in birds. The potential of novel strains with pandemic potential emerges as AIVs are able to cross species barriers and undergo genetic reassortment. The widespread host range and adaptability of H5N1 is what makes it a concern (Mekkawy et al., 2025; Purcell et al., 2025; Acharya et al., 2020).

A comprehensive management approach is needed consisting of prevention, surveillance, rapid response and vaccination. Limited movement of poultry, disinfection and proper poultry hygiene practices at the farm level are crucial to minimize transmission. Early detection and coordinated containment of the spread is also crucial, relying on surveillance systems and international cooperation (Tian et al., 2023; Tsukamoto, 2012; Debnath et al., 2023). Vaccination is a valuable long-term control measure but vaccine formulations need to be updated regularly due to the antigenic drift of the AIVs (Mekkawy et al., 2025; Thomas & Noppenberger, 2007). New tools, such as antiviral drugs and broadly protective influenza vaccines, could further enhance the ability to be ready for future outbreaks.

Also, reducing the economic impacts of AIV outbreaks can be significant through government policies. Disease control through strategic regionalization, swift removal of infected flocks, and appropriate compensation systems can help to ensure disease control and producer confidence and market stability (Swayne et al., 2017; Kim et al., 2010).

### 1.4 Major limitations of current vaccination strategies

Despite major advances in avian influenza vaccination, present vaccines often fail to induce full sterilizing immunity. Vaccinated birds may remain susceptible to infection and continue to shed virus, providing opportunities for transmission and viral evolution. Antigenic drift and reassortment further reduce protection when vaccine strains are mismatched with circulating viruses, necessitating frequent vaccine updates and limiting the feasibility of broadly protective vaccines. Inactivated vaccines may also require adjuvants and multiple doses to achieve adequate immunity, adding to the logistical burden of vaccination (Lee & Suarez, 2005; Capua & Cattoli, 2007; Swayne & Kapczynski, 2008; Fuchs et al., 2009).

There are also challenges of manufacturing and field implementation. Conventional production systems can limit the speed and flexibility of vaccine supply when new variants emerge, while maintaining adequate cold chain, high vaccination coverage and reaching smallholder poultry systems remain difficult (Johansson & Brett, 2007; Harada & Itamura, 2011; Pérez-Rubio et al., 2020; Becker et al., 2021). Therefore, successful AIV vaccination involves not only improved vaccine efficacy, but also rapid strain updating, reliable surveillance, appropriate DIVA strategies, and consistent field-level implementation.

### 1.5 Rationale, novelty, limitation and objectives of the present review

Individual vaccine technologies and immune-enhancement strategies have been considered in previous reviews, but there have been few comparisons of major vaccine platforms on the same practical criteria. We fill that gap by systematically comparing seven AIV vaccine platforms in terms of immunogenicity, protection, cost and scalability, potential for rapid updating, DIVA compatibility and major limitations (Table 1). In particular, we emphasize the gap between promising experimental results and their feasibility under field conditions.

**Table 1:** Comprehensive Comparison of All AIVs.

Vaccine Platform	Immunity Profile	Cost & Scalability	Robustness & Stability	Potential for rapid updating	DIVA compatibility	Key Limitations	Quantitative Evidence
Inactivated Vaccines	Strong humoral immunity; weak cellular immunity; 95% efficacy *vs* homologous, 78% *vs* heterologous strains (Kovács et al., 2025; Tseng et al., 2024)	Cost-effective, egg-based; well-established global infrastructure (Chai et al., 2025; Xu et al., 2023)	Stable, safe (killed pathogen); requires periodic antigen updates due to drift (Kovács et al., 2025)	Low to moderate. Requires strain selection, virus propagation and production; egg-based production can delay updating.	Moderate to high, when appropriately designed, for example heterologous NA or marker-based vaccines; conventional whole-virus vaccines are not inherently DIVA-compatible.	Adjuvants required; multiple boosters needed; antigenic drift necessitates seed strain updates (Kovács et al., 2025; Abdullatif et al., 2025)	A large meta-analysis of HPAI vaccine challenge studies provides strong quantitative evidence. Inactivated vaccines showed 95% efficacy against mortality following homologous challenge (RR = 0.05, 95% CI: 0.01–0.17) and 78% efficacy against heterologous challenge (RR = 0.22, 95% CI: 0.14–0.37) (Tseng et al., 2024).
Live Attenuated (LAIVs)	Humoral + cellular + mucosal immunity; natural infection-like response; 97% efficacy in meta-analysis (Tseng et al., 2024; Chai et al., 2025; Hui et al., 2026)	Mass application via spray or drinking water; scalable for high-density farms (Cargnin Faccin et al., 2024; Hui et al., 2026)	Reversion/recombination risk; WOAH discourages live AIV vaccines due to reassortment danger (Cargnin Faccin et al., 2024; Kovács et al., 2025)	Moderate. HA/NA can be changed through reassortment or reverse genetics, but safety and regulatory testing slow deployment.	Moderate to high, depending on marker design, but not inherent to all LAIVs.	Vaccine-related adverse events; risk of genetic reassortment generating HPAI (Kovács et al., 2025; Chai et al., 2025)	The 2024 systematic review and meta-analysis found 97% efficacy against mortality for live recombinant vaccines (RR = 0.03, 95% CI: 0.01–0.13), compared with 95% for inactivated vaccines against homologous challenge (Tseng et al., 2024).
Recombinant Subunit Vaccines	Enhanced humoral immunity; weak cellular immunity; safe, low adverse reaction risk (Kovács et al., 2025; Chai et al., 2025). *100% survival against lethal homologous H7N1 challenge with demonstrated heterologous H7N9 cross-reactive HI activity* (Falcón et al., 2024).	Expensive, complex manufacturing; requires prior antigen knowledge for design (Kovács et al., 2025; Chai et al., 2025)	Requires cold chain; adjuvants and multiple doses needed for strong immunity (Kovács et al., 2025)	High. Antigen sequences can be redesigned without propagating infectious AIV.	High potential, particularly when non-structural/marker antigens are excluded, but requires a validated companion diagnostic.	Design relies on prior virus knowledge; limited cellular immunity (Kovács et al., 2025; Chai et al., 2025)	Recombinant soluble trimeric H5 HA provided 100% protection after a single 10 µg dose; 2 µg provided 90% protection, while 0.4 µg provided 60% protection against lethal H5N1 challenge in chickens (Cornelissen et al., 2010)
Viral Vector-Based	Humoral + cellular immunity; does not interfere with maternal antibodies; long-term immunity (HVT) (Kovács et al., 2025; Tang et al., 2025)	Expensive, complex; egg-independent manufacturing; in ovo or day-of-hatch delivery (Kovács et al., 2025; Kerstetter et al., 2020)	Tolerant of HA1 variation (rHVT, RP vaccines); thermostabilizable for stockpiling (Kerstetter et al., 2020; Mo et al., 2023)	High. The inserted AIV antigen can be redesigned while retaining the vector backbone.	High potential, particularly when the vaccine antigen differs diagnostically from field infection.	Pre-existing vector immunity; species-dependent host susceptibility; virus shedding possible (Kovács et al., 2025; Tang et al., 2025)	Non-replicating adenovirus vaccination provided 68% protection against H5N1 and 100% protection against H5N2 challenge in chickens (Toro et al., 2007).
DNA Vaccines	Both humoral and cellular immunity; rapid, scalable, cost-effective production (Chai et al., 2025). *Statistically significant increase in neutralizing antibody titer (P<0.001) after HA2 DNA vaccination; challenge-protection % not reported* (*Soleimani* et al.*, 2016*)*.*	Rapid and scalable; no egg dependency; flexible antigen design (Chai et al., 2025; Dey et al., 2023)	Safety concerns; preclinical stage; delivery optimization needed (Chai et al., 2025; Mancera Gracia et al., 2020)	Very high. Plasmid sequences can be rapidly redesigned and manufactured without infectious virus.	High potential, depending on antigen/marker configuration and diagnostic assay.	Safety concerns; not yet commercialized; delivery efficiency challenges (Chai et al., 2025; Mancera Gracia et al., 2020)	DNA nanoparticle study quantitatively measured HI titers and cellular immune populations but did not provide a challenge-protection percentage. This should be explicitly stated rather than implying efficacy (Xu et al., 2023).
mRNA Vaccines	Both humoral and cellular immunity; rapid update capability; broad-spectrum potential (Dey et al., 2023). *Complete prevention of morbidity and mortality in ferrets after lethal clade 2.3.4.4b H5N1 challenge; antibody levels comparable to whole-inactivated vaccine in mice* (Furey et al., 2024).	Rapid, scalable manufacturing; no egg or cell culture needed	Newer platform; advantages in broad spectrum and flexibility	Very high. Sequence replacement is rapid and does not require propagation of the new virus strain.	High potential, because the vaccine can encode selected antigens rather than the complete virus.	Research gaps in immune persistence and accessibility; preclinical for AIV (Dey et al., 2023)	In SPF chickens, two 5 µg doses initially produced 100% homologous protection and 50% heterologous protection; optimized SY2-HA mRNA-LNP subsequently achieved 100% protection against both homologous and heterologous H5N1 strains (Wang et al., 2025).
Virus-Like Particles (VLPs)	Strong humoral + cellular immunity; safe, no infectious material (Kovács et al., 2025; Nuwarda et al., 2021). *100% protection against lethal H9N2 challenge in mice and ferrets* (*Pushko* et al.*, 2005*; *Pushko* et al.*, 2007*)*.*	Expensive, complex manufacturing; long development time (Kovács et al., 2025)	Requires adjuvant/booster for long-term immunity; cold chain needed (Kovács et al., 2025)	High. HA/NA composition can be redesigned or multivalent formulations generated.	Moderate to high, depending on antigen composition and accompanying diagnostic strategy.	High production cost limits large-scale application; specific storage required (Kovács et al., 2025)	H5 VLP vaccination studies in chickens reported 100% protection (11/11 chickens survived, no clinical signs) after lethal homologous H5N1 challenge in chickens immunized with HA-VLP or HAM-VLP Challenge virus shedding was reduced to only 2/11 (HA-VLP) and 1/11 (HAM-VLP) birds via the oropharyngeal route at 5 dpc, *versus* 0/11 via the cloacal route No virus detected in brain, trachea, lung, spleen, kidney or cecal tonsil (0/3 tested) in either VLP group By contrast, all unvaccinated controls died at 2–3 dpc with virus present in every organ tested (titers 10³·⁴–10⁶·⁰ TCID₅₀/0.1 ml) (Choi et al., 2013).

**Key Definitions:**.

**Antigenic drift** refers to the gradual accumulation of mutations, particularly in viral surface proteins, that can modify antigenic recognition (Cattoli et al., 2011; Cox & Karl Anderson, 2007).

**Antigenic shift** refers to the abrupt generation of novel influenza viruses through reassortment of genome segments between different influenza viruses (Balish et al., 2010; Epstein & Price, 2010).

**Highly pathogenic avian influenza (HPAI)** is an infection with avian influenza virus (AIV) that causes severe systemic disease and high mortality in domestic poultry, especially chickens and turkeys. HPAI viruses are characterized mainly by their ability to cause a high mortality rate in experimentally infected chickens and/or by the presence of a polybasic cleavage site in the hemagglutinin (HA) protein, which permits efficient cleavage of HA by ubiquitous host proteases and enables systemic viral replication (Swayne, 2007; Alexander, 2007).

**Low-pathogenic avian influenza (LPAI) meaning that** naturally circulating viruses usually cause mild or subclinical infections in poultry and lack the molecular characteristics associated with high pathogenicity, such as a polybasic HA cleavage site (Swayne, 2007; Alexander, 2007) .

**DIVA** refers to vaccination and diagnostic strategies designed to differentiate infected animals from vaccinated animals (Capua et al., 2003; Capua et al., 2005).

DIVA compatibility should not be treated as an intrinsic property of an entire vaccine platform. It depends on the antigen composition, marker strategy, and companion diagnostic assay. So instead of using Yes or NO we have used terms such as “high potential,” “moderate potential,” or “design-dependent”.

**Broadly neutralizing antibodies (bNAbs)** are antibodies capable of recognizing conserved viral epitopes and neutralizing multiple antigenically distinct influenza viruses (Temperton et al., 2007; Tsai et al., 2009; Clementi et al., 2012).

Previous reviews of AIV vaccination were published before the rapid worldwide dissemination of H5 clade 2.3.4.4b in 2020–2022. This lineage has spread to multiple continents and has increasingly involved mammalian hosts since 2021, thus altering the priorities for AIV vaccine development and application (Xie et al., 2023; Kandeil et al., 2023). These developments highlight the need for easily updatable vaccines, that limit viral shedding, provide broad protection, support DIVA strategies and are still practical and affordable for large scale poultry production.

This review is a narrative synthesis rather than a formal systematic scoping review, and several limitations follow from that approach. First, no formal risk-of-bias or study-quality assessment was applied to individual studies; findings from small preclinical challenge studies are therefore presented alongside larger or field-validated studies without a standardized quality weighting. Second, the search, though conducted across four databases with a structured Boolean strategy (Section 1.6), was not registered and did not follow the full PRISMA-ScR reporting checklist, so study selection could be subject to selection bias despite our efforts to search broadly and screen reference lists of key reviews. Third, the evidence base is weighted toward preclinical (mouse, ferret, or small-scale poultry challenge) studies for next-generation platforms (recombinant subunit, viral-vector, DNA, mRNA, VLP), with comparatively fewer large-scale field-validated data than for inactivated and live-attenuated vaccines; this asymmetry is itself one of the review's central findings (Section 6) rather than a methodological oversight, but it does mean that platform comparisons in Table 1 should be interpreted as reflecting current evidence maturity as well as intrinsic platform performance. No formal publication-bias assessment (e.g., funnel plot) was performed, consistent with the narrative rather than meta-analytic design (see also Section 1.6).

Recent developments in artificial intelligence (AI) and machine learning (ML) are also predicted to help develop and optimize next-generation avian influenza vaccines. A recent bibliometric study about the application of AI/ML in veterinary and animal sciences showed that this research area has experienced a significant growth over the past few years, moving beyond conventional machine-learning methods to deep learning, predictive modeling and animal-health/disease-diagnosis/data-driven applications (Ali et al., 2026). These computational methods could be important in the integration of large-scale genomic, antigenic, immunological and epidemiological data to aid in antigen selection, viral evolution prediction, the identification of conserved protective epitopes and rational vaccine design. Like any nascent technology, however, their use in avian influenza control will need high-quality datasets, models that can be explained, field testing, and collaboration among international producers to make them applicable to a variety of production systems and geographic areas (Ali et al., 2026).

Section summary. The constant evolution of avian influenza viruses and the limitations of strain-matched conventional vaccines underline the need for broader, more durable and adaptable vaccination strategies in general. While traditional vaccines will continue to have a role in reducing disease and mortality, the antigenic variability, incomplete control of infection and viral shedding, and constraints in manufacturing and deployment of vaccines limit their ability to provide broad protection against emerging strains. These challenges provide the rationale to evaluate next generation platforms that can improve breadth, flexibility and immune durability including recombinant subunit, viral-vector, DNA, mRNA and virus-like particle (VLP) vaccines.

### 1.6 Literature search and study selection

A comprehensive literature search was conducted to identify published evidence on the development, evaluation, application, and future prospects of vaccines against avian influenza viruses. Searches were performed in PubMed/MEDLINE, Scopus, Web of Science Core Collection, and Google Scholar, with additional relevant publications identified by screening the reference lists of key articles and recent reviews.

The search strategy combined controlled vocabulary and free-text terms related to avian influenza, vaccination, vaccine platforms, immunogenicity, protection, delivery, and field application. The principal search terms included:

("avian influenza" OR "avian influenza virus" OR AIV OR HPAI OR H5N1 OR H5N8 OR H7N9 OR H9N2) AND (vaccine OR vaccination OR immunization) AND (inactivated OR "live attenuated" OR recombinant OR "subunit vaccine" OR "viral vector" OR adenovirus OR HVT OR DNA OR mRNA OR "virus-like particle" OR VLP) AND (immunogenicity OR efficacy OR protection OR "viral shedding" OR transmission OR DIVA OR "mucosal immunity" OR "cross-protection" OR scalability OR stability OR delivery OR manufacturing).

Additional searches were conducted using combinations of terms related to antigenic drift and shift, conserved epitopes, broadly neutralizing antibodies, universal influenza vaccines, mosaic antigens, nanoparticle vaccines, mucosal vaccination, prime-boost vaccination, *in ovo* vaccination, hatchery vaccination, spray vaccination, and mass vaccination. The search strategy was adapted to the syntax of each database. The search covered literature published from January 2000 to June 2026, with particular emphasis on studies published during the most recent decade.

Studies were considered relevant when they addressed at least one of the following: (i) development or evaluation of an AIV vaccine platform; (ii) immune responses and protective efficacy in poultry or relevant animal models; (iii) reduction of viral replication, shedding, or transmission; (iv) antigenic breadth or cross-protection; (v) vaccine delivery and manufacturing; (vi) DIVA compatibility; or (vii) practical, regulatory, and economic considerations affecting implementation in poultry production. Both original research articles and relevant reviews, meta-analyses, and authoritative reports were considered. Publications unrelated to AIV vaccination, studies lacking sufficient information on vaccine characteristics or outcomes, and duplicate publications were excluded.

Particular emphasis was placed on recent literature addressing next-generation vaccine technologies and current AIV lineages, while foundational studies were retained when they provided essential evidence regarding established vaccine platforms. The retrieved evidence was narratively synthesized and comparatively evaluated according to immunogenicity, safety, antigenic flexibility, duration of protection, reduction of viral shedding and transmission, DIVA compatibility, delivery feasibility, manufacturing requirements, cost, scalability, and field applicability. This comparative framework was used to identify the principal technological gaps and to propose priorities for future AIV vaccine development. The manuscript itself emphasizes that these practical criteria are essential because strong experimental immunogenicity alone does not establish suitability for commercial poultry production. This review is intended for researchers developing avian influenza vaccines, veterinary virologists, poultry health specialists and regulatory scientists.

## 2 Biology of avian influenza virus relevant to vaccine development

### 2.1 Virus structure and antigenic sites critical for neutralizing immunity

The two major surface glycoproteins of AIVs hemagglutinin (HA) and neuraminidase (NA) are essential for viral entry, replication and immune evasion (Xu et al., 2010; Böhm et al., 2014; Krause et al., 2014; Russell, 2016; Byrd-Leotis et al., 2017). HA mediates attachment and fusion with host cells, and NA mediates release of newly formed virions, thus promoting viral propagation (Krause et al., 2014; Byrd-Leotis et al., 2017). These glycoproteins are the main targets for neutralizing antibodies and vaccine development and have a strong influence on viral host range, pathogenicity and antigenic properties.

HA is a type I membrane glycoprotein that is synthesized as a precursor (HA0), cleaved into HA1 and HA2 subunits, and assembled into a homotrimer. The HA1 subunit makes up the globular head of the protein, containing the receptor-binding site (RBS), while the HA2 subunit forms the stem region that mediates membrane fusion (supplementary Figure 3.1). HA binds to sialic acid receptors on the host cell. Avian influenza viruses have a preference for α2,3-linked sialic acids, whereas mammalian viruses prefer α2,6-linked receptors. This receptor specificity plays a key role in host adaptation and interspecies transmission (Byrd-Leotis et al., 2017). HA is also the main antigenic determinant of influenza viruses, which undergo continuous antigenic drift mainly in the globular head region to escape immune response and prevent vaccine development (Russell, 2016; Byrd-Leotis et al., 2017). There are 16 identified HA subtypes (H1-H16), which play a role in the diversity and emergence of novel strains of influenza (Fereidouni et al., 2023). In contrast, the conserved HA stem region is less variable and represents a promising target for universal influenza vaccines. Broadly neutralizing antibodies (bnAbs) to this region can protect against multiple AIV subtypes (Zhao et al., 2022). The RBS within the HA globular head is a major focus for neutralizing antibodies. The antigenic surface of H5N1 HA involved in antibody recognition is formed by residues like Ser^126^, Lys^165^, Arg^166^, Ser^167^, Tyr^168^, Asn^169^, Thr^171^, and Asn^172^ (Lin et al., 2018; Wang et al., 2019). Mutations in these regions can reduce antibody binding and allow immune escape. For example, substitutions at positions 144–147 within antigenic site A have been associated with resistance to neutralizing antibodies, highlighting the importance of antigenic surveillance and epitope-focused vaccine design (Okuda et al., 2019).

NA is a receptor destroying enzyme that cleaves off sialic acids from the surface of host cells and viral glycoproteins, thus preventing virion aggregation and promoting release of the virus (supplementary Figure 3.2) (Böhm et al., 2014; Russell, 2016; Byrd-Leotis et al., 2017; Halbherr et al., 2015). Nine NA subtypes (N1-N9) contribute to the influenza antigenic diversity (Echeverry & Rodas, 2011). NA is also the target of antiviral drugs such as oseltamivir and zanamivir, and NA-specific antibodies can inhibit viral spread, thus supporting its role as a complementary vaccine antigen (Gubareva & Mohan, 2022).

Other conserved proteins such as matrix 2 (M2) and nucleoprotein (NP) (Supplementary Fig. 3.3) (Anwar et al., 2006) are investigated as alternative targets for next-generation influenza vaccines. (Supplementary Table 1 and Fig. 2) illustrated the contribution of HA and NA antigenic variation, immune escape mechanisms and conserved viral structures to the development of broadly protective vaccines and antiviral strategies.

**Fig. 2:**
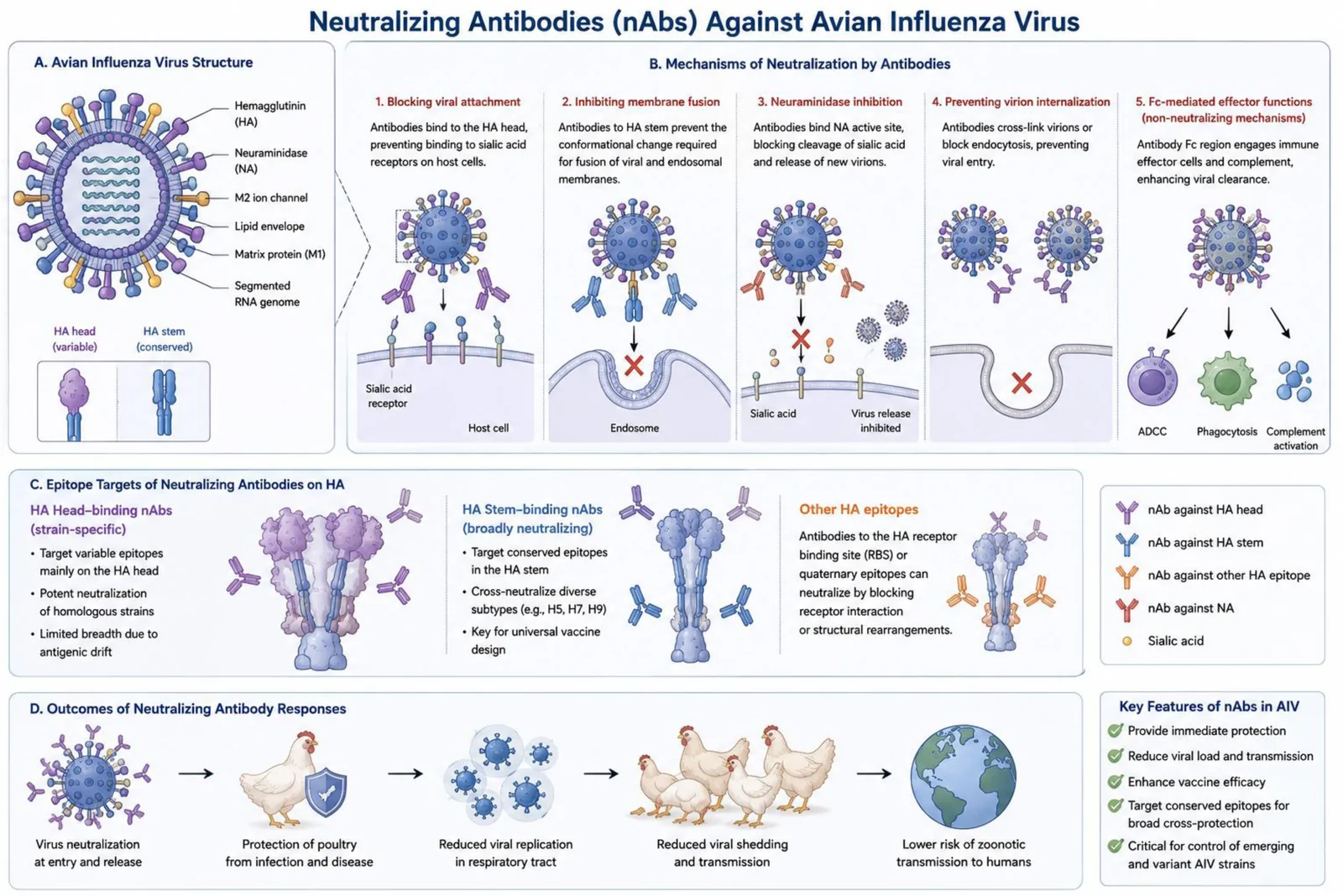
Mechanisms and protective functions of neutralizing antibodies (nAbs) against avian influenza virus (AIV). The schematic illustrates the major targets and mechanisms through which nAbs interfere with AIV infection: (A) AIV structure and antibody targets: the major viral surface proteins, hemagglutinin (HA) and neuraminidase (NA), are highlighted, with the variable HA head and relatively conserved HA stem representing important antibody targets; (B) Mechanisms of antibody-mediated neutralization: antibodies can block HA-mediated attachment of the virus to sialic acid receptors on host cells, interfere with HA-mediated membrane fusion, inhibit NA enzymatic activity and viral release, and prevent virion internalization. Fc-dependent effector mechanisms, including antibody-dependent cellular cytotoxicity (ADCC), phagocytosis, and complement activation, can further contribute to viral clearance; (C) Major antibody epitopes: HA head-directed antibodies generally provide potent but strain-specific neutralization, whereas antibodies targeting conserved HA stem epitopes have greater potential for cross-neutralization among antigenically diverse AIV strains. Additional neutralizing epitopes within HA can interfere with receptor binding or structural rearrangements required for infection; (D) Protective outcomes: neutralizing antibody responses can inhibit viral infection and replication, reduce viral load and shedding, limit transmission among poultry, and contribute to protection against AIV disease. Targeting conserved viral epitopes is particularly relevant to the development of broadly protective and next-generation AIV vaccines.

### 2.2 Genetic diversity and evolution; antigenic drift and antigenic shift

The genetic and antigenic diversity of AIVs is due to continuous evolutionary processes that enable viral adaptation, immune escape and emergence of novel strains (Kalthoff et al., 2013). These changes are mostly caused by antigenic drift and antigenic shift, gradual and abrupt mechanisms of influenza virus evolution respectively. In addition to the segmented nature of the viral RNA genome, these processes complicate disease control, vaccine development, and long-term prevention strategies (Ferguson et al., 2003; Huang & Yang, 2011).

**2.2.1. Antigenic drift** occurs by the accumulation of point mutations resulting from the error-prone viral RNA polymerase, particularly in the surface glycoproteins HA and NA. Mutations in antigenic regions can modify antibody recognition so that newly emerged variants can escape existing immunity and outcompete previously circulating strains (Ferguson et al., 2003). For instance, substitutions A^125^T, A^151^T and L^217^Q in H7N9 viruses correlate with lower hemagglutination inhibition (HI) titers, indicative of antigenic diversification and reduced antibody reactivity (Chang et al., 2019). These ongoing changes contribute to viral persistence in avian reservoirs, recurrent outbreaks in poultry and amplified opportunities for zoonotic transmission.

**2.2.2. Antigenic shift**, however, is caused by reassortment of genome segments between genetically different influenza viruses. Since AIVs have a segmented RNA genome (Pawestri et al., 2020; Zhao et al., 2025), co-infection of a host with different viral strains can lead to the generation of reassortant viruses with new antigenic properties. In the past, such occurrences have helped to generate pandemic strains of influenza. The evolution of H5N1 and H7N9 demonstrates the importance of reassortment and adaptive mutations in the HA receptor-binding region, which enhanced recognition of human-type receptors and enabled zoonotic infections associated with severe disease outcomes (Zhao et al., 2025).

Ecological and host factors also play a large role in the evolution of AIVs. The main reservoirs of influenza A viruses are wild aquatic birds, particularly members of the orders Anseriformes (ducks and geese) and Charadriiformes (gulls and shorebirds), and they contain the full complement of HA and NA subtypes (Fig. 1). Flyways also allow migratory birds to spread viral lineages throughout the world and to introduce new variants into domestic poultry populations. Environmental factors like temperature, precipitation, and habitat features also affect virus survival and transmission dynamics.

The rapid evolution of AIVs has direct consequences for disease management. For example, despite vaccination efforts, H5N1 viruses in Egypt have continued to diversify into multiple antigenic variants, resulting in reduced vaccine effectiveness and complicating outbreak control (Watanabe et al., 2012; Cattoli et al., 2011). Likewise, the emergence of distinct antigenic groups of H9N2 in China challenged existing vaccination strategies and emphasized the need for continuous vaccine evaluation and updating (Zhu et al., 2024).

**Section summary.** The literature identified in this review demonstrates substantial diversification of avian influenza vaccine research beyond conventional inactivated formulations. Evidence was therefore synthesized across vaccine platforms, experimental models, immune responses, protection outcomes, scalability, stability, DIVA potential, and the capacity for rapid vaccine updating. Because this review is designed as a narrative synthesis rather than a formal systematic review or meta-analysis, the available evidence was interpreted in the context of differences in experimental design, host species, vaccine formulations, challenge viruses, and reported endpoints.

## 3 Conventional avian influenza vaccines

### 3.1 Inactivated vaccines; production methods (egg-based, cell-culture), adjuvants and formulations

Following a presentation of the rationale and drawbacks of current avian influenza vaccination, this section reviews conventional vaccine platforms and their established roles in disease control, providing the foundation for consideration of next-generation alternatives.

The production of inactivated AIV vaccines is mainly based on two platforms: embryonated chicken eggs and cell culture systems. For decades the traditional method has been egg-based, where the virus is propagated in embryonated eggs and then harvested, purified and inactivated, to make the final vaccine product (Gregersen, 2008; Manini, 2017). This method has been in use successfully for a long time and has well established regulatory pathways but has several limitations. Vaccine production can take nearly six months from strain selection to distribution, and virus growth efficiency may vary considerably among influenza strains. Furthermore, large outbreaks in poultry can impact the availability of eggs and biosecurity concerns during pandemics (Gregersen, 2008; Manini, 2017; Nazari et al., 2018).

Although mammalian cell culture systems such as MDCK and Vero cells have become established platforms for human influenza vaccine production, their application in commercial poultry vaccines remains limited. Currently, most licensed avian influenza vaccines for poultry continue to be produced using embryonated chicken eggs. Cell-culture platforms therefore came out as a more flexible alternative, by using mammalian cell lines such as Madin-Darby canine kidney (MDCK), Vero and HEK-293 cells for virus propagation (Kistner et al., 2007; Howard et al., 2008; Hu et al., 2008; Barrett et al., 2010; Le Ru et al., 2010; Plosker, 2012; Schuind et al., 2015). These systems provide a faster response to emerging influenza strains, allow more predictable production yields, and reduce dependence on poultry resources. Their scalability and lower risk of contamination with avian pathogens make them increasingly valuable for sustainable vaccine manufacturing and pandemic preparedness (Barrett et al., 2010; Plosker, 2012). (Supplementary Table 4).

Adjuvants are added to inactivated vaccines to increase immune stimulation and extend duration of immunity. Aluminum hydroxide is still one of the most widely used adjuvants and can induce strong antibody responses and protection in animal models (Chang et al., 2010). Chitosan is a natural polymer from chitin, which also improves the immune responses but usually has lower antibody titers than oil-based adjuvants (Chang et al., 2010). Oil-based emulsions generally give the strongest antibody responses among the available formulations and have shown high protective efficacy against poultry (Lone et al., 2017). Montanide™ adjuvants, such as ISA71 and Gel02, have been successfully employed by injection or spray delivery to enhance humoral and cellular immunity against H5N1 and H9N2 virus, especially in prime-boost vaccination strategies (El Naggar et al., 2017; Ismail et al., 2018).

Advanced adjuvants have further improved influenza vaccine efficacy by enhancing both humoral and cellular immunity. Oil-in-water emulsions such as MF59 and AS03 strengthen immune responses, particularly in populations with reduced immune function (Atmar & Keitel, 2009; Del Giudice & Rappuoli, 2014; Wong et al., 2017). CpG oligodeoxynucleotides (CpG ODN) activate Toll-like receptor 9 and promote strong antibody responses, including IgM and IgY production (Singh et al., 2015). Other formulations, including synthetic peptide adjuvants, AddaVax, and PolyI, have increased HI titers and broadened antibody responses compared with conventional aluminum-based adjuvants (Xiao et al., 2017; Nian et al., 2021; Gao et al., 2022).

Natural compounds such as naringenin, lovastatin and β-glucans enhance B-cell activation, germinal center formation and mucosal immunity against H5 subtype viruses (Jiao et al., 2025; Song et al., 2025). Several adjuvants also stimulate cellular immunity by promoting T-cell activation, cytokine production and balanced Th1/Th2 responses. For example, naringenin and lovastatin enhance CD^4+^ and CD8^+^ T-cell responses and IFN-γ secretion, and MF59 with CpG improves Th1-biased immune responses (Wack et al., 2008; Cheong et al., 2021). Combinations like MF59 with CpG further potentiate Th1-biased immunity, characterized by elevated IgG2a levels and stronger interferon responses (Wack et al., 2008). Chicken immune modulators (cIFITM1, cIFITM3 and cViperin) derived from chickens also reduce viral shedding and tissue damage. Formulations based on AMP-ZnO nanoparticles increase mucosal IgA responses, goblet cell activity, and cytokine expression, thus increasing barrier protection in vaccinated birds (Liu et al., 2024; Liu et al., 2024).

Vaccine effectiveness also depends greatly on the delivery route. Intramuscular vaccination remains the standard method for inducing systemic immunity, particularly when combined with potent adjuvants (Lone et al., 2017; Singh et al., 2015). In contrast, intranasal and spray-based approaches promote mucosal immunity and are attractive for large-scale poultry vaccination programs (El Naggar et al., 2017). Encapsulation technologies, including PLGA nanoparticles carrying CpG ODN, provide combined systemic and mucosal protection, while prime-boost strategies can strengthen immune memory (Ismail et al., 2018; Singh et al., 2016). Cationic polymers such as GenJet™ and in vivo-jetPEI® further improve antigen delivery and stimulate balanced humoral and cellular responses (Cao et al., 2018) (Supplementary Table 5).

### 3.2 Live attenuated vaccines (LAIVs); strategies of attenuation and safety concerns

LAIVs are important approaches for controlling influenza outbreaks in poultry and reducing the risk of zoonotic transmission. Their development depends on achieving sufficient attenuation while maintaining replication capacity and strong immunogenicity. Two major strategies, temperature-sensitive (TS) mutations and targeted gene deletion, have been widely used to generate safe and effective LAIV candidates (Supplementary Figure 4).

Temperature-sensitive mutations are among the most established attenuation strategies for LAIV development. These mutations permit viral replication at lower temperatures but restrict replication at higher body temperatures, resulting in reduced virulence (Supplementary Table 6). Polymerase gene modifications, particularly in PB1 and PB2, play a central role in this phenotype. Mutations such as PB1 (391E, 581 G, 661T) and PB2 (265S), together with NP (34 G) substitution, have been associated with strong attenuation and temperature sensitivity (Zhang et al., 2012; Isakova-Sivak et al., 2011; He et al., 2013; Broadbent et al., 2014). The generation of 6:2 reassortant viruses, containing six internal genes from a cold-adapted master donor virus and two surface antigen genes (HA and NA) from circulating strains, enables LAIVs to preserve attenuation characteristics while displaying relevant antigenic profiles (Broadbent et al., 2014; Kiseleva et al., 2011).

Differences in RNA secondary structures, particularly within nonstructural protein 2 (NS2), PA, PB2, and NP mRNAs, may also contribute to cold adaptation and temperature sensitivity (Chursov et al., 2012). Additional safety improvements have been achieved by incorporating microRNA response elements (MREs) into viral genomes, allowing targeted suppression of viral replication and enhanced immune responses (Perez et al., 2009). Mutations that increase polymerase fidelity, such as PB1-L^66^V, further improve genetic stability and reduce the possibility of reversion to virulence (Mori et al., 2021).

Gene deletion is another widely used attenuation strategy. The deletion or truncation of the NS1 gene reduces viral pathogenicity by impairing the ability of influenza viruses to suppress host interferon responses. NS1-modified LAIVs have demonstrated strong immunogenicity, reduced disease severity, and cross-protection against different influenza clades (Pica et al., 2011). Sublingual administration of NS1-deleted strains, including ΔNS1 H1N1 and ΔNS1 H5N1, has induced both mucosal and systemic immune responses, while engineered NS1 truncations further enhanced interferon activation and vaccine efficacy (Park et al., 2012).

Combining multiple attenuation strategies has improved LAIV performance by increasing genetic stability and protective efficacy. LAIVs incorporating TS mutations, targeted deletions, or miRNA-based attenuation have demonstrated strong protection against influenza challenge in animal models (Gao et al., 2020). These vaccines induce comprehensive immune responses, including mucosal IgA, systemic antibodies, and cellular immunity, which contribute to broader protection against diverse avian influenza strains (Zheng et al., 2012). Despite these advantages, safety concerns remain major challenges in LAIV development. The primary risks include reversion to virulence and reassortment with wild-type influenza viruses (Hai et al., 2011; Kenney et al., 2011). Chimeric vaccine constructs and application strategies such as in *ovo* vaccination provide additional safety benefits by inducing early immunity and reducing exposure of susceptible populations to live vaccine viruses (Ghorbani et al., 2023). (Supplementary figure 1)

Despite these immunogenic advantages, intranasal and spray-delivered LAIVs have not achieved wide commercial deployment against AIV, for reasons that are largely regulatory and safety-driven rather than immunological. WOAH guidance discourages the use of live AIV vaccines in poultry because of the risk of reversion to virulence and reassortment with field strains circulating in the same flock (Cargnin Faccin et al., 2024; Kovács et al., 2025), and national regulators have generally required a level of genetic-stability evidence for LAIV candidates that is more difficult to generate at commercial scale than for inactivated vaccines. Field experience with other intranasally delivered poultry LAIVs (*e.g.*, against LPAI H9N2) illustrates the practical trade-off: strains attenuated enough to satisfy safety requirements have sometimes shown reduced or inconsistent protective efficacy in the field, while strains retaining strong immunogenicity have raised reversion concerns, complicating regulatory approval (Pica et al., 2011; Park et al., 2012). As a result, most licensed AIV vaccination programs continue to rely on inactivated or vector-based platforms for routine use, reserving LAIV-type approaches largely for experimental or emergency settings.

### 3.3 Recombinant subunit vaccines and production platforms

Recombinant subunit vaccines are very safe and targeted vaccines, as they utilize selected viral antigens, including HA, NA and M2, instead of whole infectious virus, to vaccine against avian influenza. They have been studied in insect cell, plant, yeast and bacterial systems, and each system has its own merits with regards to antigen production, scalability, and immunogenicity.

The baculovirus expression vector system (BEVS) has emerged as a promising platform for producing recombinant HA. Using the CrisBio® system, Falcón et al. (2024) (Falcón et al., 2024) produced purified H7N1 HA at ∼75 mg/L from Trichoplusia ni pupae. Chickens vaccinated with both the monomeric and trimeric HA formulation received two 20 μg doses and were subsequently challenged with a highly virulent H7N1 strain. All birds vaccinated (11/11 in each group) survived the 11-day challenge period without clinical signs, whereas none of the mock-vaccinated controls survived, indicating 100% survival and full protection against lethal homologous challenge. Both formulations induced HA specific IgY titres >1:2000 and HI titres to H7N1 were above 1:40. Importantly, the sera also demonstrated HI activity to heterologous H7N9 virus, indicating measurable cross-reactive antibody responses (Falcón et al., 2024).

Further antigen engineering revealed that the full-length, membrane bound HA (mH7) was more stable and immunogenic than the soluble HA ectodomain (sH7), underscoring the critical need to retain the native antigen structure (Chia et al., 2019). An alternative platform that may be scalable for the production of recombinant influenza vaccines is plant-based expression systems. Several methods have been employed to generate HA and M2 antigens for use in inducing immune responses, including both transient and stable expression (Mardanova & Ravin, 2018). Plant systems are also appealing for vaccine manufacturing and rapid antigen production due to their relatively simple production requirements, and the lack of animal-derived pathogens (Ceballo et al., 2018).

In addition to the benefit of eukaryotic post-translational modification, yeast expression systems, *Pichia pastoris* and *Saccharomyces cerevisiae*, possess the added advantage of being easy to grow. In preclinical studies, Pichia pastoris has been used to express HA proteins with high yields and high immunogenicity (Baghkheirati et al., 2024). Likewise, *Saccharomyces cerevisiae* has been used to formulate HA-based recombinant protein vaccines and novel DNA/RNA hybrid vaccines, which have both shown to be effective in inducing both humoral and cell-mediated responses and protection against H9N2 challenge in poultry (Zhang et al., 2023). The results demonstrate that yeasts are potential hosts to produce high-quality antigens for commercial production of vaccines.

Bacterial systems especially *Escherichia coli* (*E. coli*) is a fast and economical system for producing recombinant antigens. Even though *E. coli* is not a eukaryotic cell, antigen design and fusion strategies can be used to enhance its immunogenicity. For instance, an HA1-2-flagellin (fliC) fusion protein induced high levels of HA specific antibodies and neutralizing responses in animal models (Song et al., 2015).

Although they have these benefits, recombinant subunit vaccines are not as immunogenic as whole-virus vaccines and may need the proper adjuvants or delivery systems. Other factors to consider when maintaining vaccine efficacy include antigen stability and preservation of conformational epitopes. Enhancement of the design and expression of these antigens, as well as formulating them into an effective and feasible subunit vaccine to control avian influenza, therefore requires further optimization.

*Section summary:* Traditional inactivated and live attenuated influenza vaccines continue to be important tools in controlling avian influenza and can provide substantial protection against disease and death if the vaccine and circulating strains are well matched. Their effectiveness, however, may be limited by antigenic mismatch, variation in immune responses, incomplete prevention of infection or viral shedding and limitations related to production, administration and updating. These limitations, along with the continuing emergence of genetically and antigenically divergent viruses, support the development of alternative platforms that can induce broader immunity and be more rapidly adapted to new strains as they emerge.

## 4 Next-Generation avian influenza vaccines

Building on the strengths and limitations of conventional vaccines, this section focuses on next-generation platforms designed to improve immunogenicity, adaptability, breadth of protection, and vaccine-update capacity.

### 4.1 Viral vector-based vaccines

Viral vector-based vaccines are promising vaccine platforms and can be used for the development of vaccines for AIV, especially for highly pathogenic avian influenza viruses. The influenza vaccines are based on recombinant viral vectors containing influenza antigens, primarily HA, and they can elicit both humoral and cellular immune response (supplementary Fig.5).

HA gene from H5N1 and other AIV strains has been efficiently expressed in adenoviral vectors that have demonstrated good immunogenicity and protective efficacy, and even cross-protection against antigenically related influenza viruses (Zhang, 2012). Due to they are not capable of replication, they can be produced relatively quickly, and they are scalable, they are attractive for the development of vaccines to be rapidly adapted during AIV outbreaks.

There has also been some research looking into whether herpesvirus of turkey (HVT) could be a vector for AIV vaccines. Recombinant HVT vaccines expressing the HA of AIV can induce long lasting immunity, prevent clinical disease in vaccinated birds and reduce shedding of infectious virus from vaccinated birds (Abd El-Hamid et al., 2018; Criado et al., 2023). The HVT is widely used in poultry vaccination programs, therefore the HVT-based AIV vaccines could be incorporated in the present vaccination program, which is a feasible solution for the field.

Viral vector vaccines can also induce mucosal immunity, especially against respiratory diseases like AIV. Improved mucosal responses may help limit virus replication and transmission in the poultry population (Ma et al., 2024). Their effectiveness, however, may be affected by vector-specific immunity, antigenic matching, vaccine dose, and route of vaccine administration. Thus, optimization of vaccine delivery and antigen expression still remains to be important for providing consistent protection in the field.

Fowlpox virus also has been used as a vector for AIV antigen delivery. A recombinant fowlpox virus carrying the H5 HA gene that has been shown to provide protection against H5 virus in vaccinated birds and to reduce viral shedding after challenge (Bublot et al., 2006) is being developed as the TROVAC—H5 vaccine. This will give another vector system for making recombinant vaccines against HPAIV. The other approach is to make it more immunogenic to produce recombinant influenza antigens. The αGal HyperAcute™ technology is the combination of αGal epitopes with recombinant proteins to increase immune recognition and has been studied as a strategy for H5N1 and H7N9 vaccine candidates (Chen et al., 2017).

Viral vector-based vaccines have some potential benefits for AIV control and include efficient delivery of the antigen, induction of cellular and humoral immunity, long-lived immunity and suitability for poultry vaccination. When considering the development of an effective field vaccine against AIV, however, several factors are important to consider including vector immunity, antigenic variation, vaccine delivery and the ability to reduce viral shedding.

### 4.2 DNA vaccines mechanisms of action and delivery strategies

DNA vaccines are a promising next generation vaccination strategy for AIV. They express antigens, such as HA of influenza *in situ* using plasmid DNA. This creates an antigen which is processed and presented to the immune system, causing humoral and cell-mediated immune response. The relatively simple design, rapid production, and lack of live virus in DNA vaccines makes them attractive for the development of rapidly deployable AIV vaccines (Soleimani et al., 2016). Strong antibody responses are induced to influenza antigens upon DNA vaccination. For instance, antibody titers of immunized animals have been found to have been elevated after immunization with HA2 based DNA vaccines, showing their immunogenicity (Ma et al., 2024).

Molecular and chemical adjuvants can be used to further enhance this response. CCL19 plays a role in stimulating antibody formation and attracting lymphocytes, while α-galactosylceramide (α-GalCer) activates natural killer T (NKT) cells to stimulate IgG responses (Fotouhi et al., 2017; Xiang et al., 2024).

The added advantage of the use of nano-adjuvants is that they can protect DNA from degradation and can augment antigen delivery, which would lead to enhanced hemagglutination-inhibition activity and antibody responses (Ma et al., 2022). DNA vaccines also induce cell-mediated immunity, including CD^8+^ cytotoxic T lymphocytes (CTLs) and interferon gamma (IFN-γ) (Best et al., 2009) that form part of the mechanisms leading to the elimination of infected cells and more sustained protection.

Significant enhancement in antigen expression and uptake of DNA has been achieved by electroporation, leading to increased production of IFN-γ and proliferation of antigen-specific T cells (Best et al., 2009). These responses are likely to be further stimulated with the use of CCL19 as a molecular adjuvant, by promoting immune-cell recruitment to the vaccination site (Xiang et al., 2024).

How and which way the DNA is introduced is crucial for the effectiveness of vaccines. Intradermal delivery may result in greater antibody responses, while intramuscular delivery tends to elicit cellular immune responses. Thus, the delivery routes could be combined to get a more balanced immune response (Lin et al., 2011).

One of the applications of particular interest for poultry production is *in-ovo* vaccination, where DNA vaccines can be introduced to the egg prior to hatching, thereby not requiring to handle individual newly hatched chickens (Laddy et al., 2009; Livingston et al., 2010; Lin et al., 2012; Amante et al., 2015).

Two promising strategies that were developed to achieve better delivery of DNA vaccines are electroporation and Nanoparticles. The transient increase of permeability of the cell membrane through electroporation increases the amount of plasmid uptake and antigen expression. Immunity can be enhanced with low voltage without causing tissue damage (Lin et al., 2012). DNA can be protected from enzymatic degradation and internalized by antigen-presenting cells using nanocarriers such as dendritic polylysine (DGL) polymers and manganese containing liposomes (Ma et al., 2022; Xu et al., 2023; Xu et al., 2023). These systems may also increase antigen presentation and lymphocyte activation, which would lead to more potent antibody and T-cell responses than would be generated with naked DNA. Overall, the DNA vaccine platform is very versatile for AIV vaccination and can be used to design antigens rapidly and can induce both humoral and cellular immunity. Efficacy can be further enhanced by optimization of antigen selection, by adding adjuvants, by electroporation, administration of nanoparticles and by *in ovo* administration. The ability to develop scalable delivery systems and formulations is also likely to be key to the practical application of these approaches to poultry vaccination.

### 4.3 mRNA vaccines: emerging applications in poultry, stability and delivery challenges in veterinary settings

The mRNA vaccine platform is a novel vaccine platform for AIV vaccination. They employ synthetic messenger RNA encoding influenza antigens which are translated directly in host cells to generate the target antigen and stimulate humoral and cellular immune responses without the use of live or attenuated virus (Hatta et al., 2024). The order-based design of their vaccines enables the ability to easily change vaccine candidates if a new or antigenically variant AIV strain emerges (Hatta et al., 2024; Leonard et al., 2025; Wang et al., 2025; Xu et al., 2023; Cheng et al., 2024) Self-amplifying mRNA (sa-mRNA) vaccines are another advancement of this technology. With the addition of replicase elements, sa-mRNA can replicate the RNA template inside the cells to maintain antigen expression. This can lead to greater immunogenicity and/or lower requirement for RNA per dose, which is crucial for large-scale poultry vaccination (Snoeck et al., 2023; Snoeck et al., 2026)

Experimental studies in avian and mammalian models have demonstrated that AIV mRNA vaccines can lead to robust antibody and cellular immune response, such as high neutralizing antibody titers and activation of cytotoxic T lymphocytes. Several studies have reported protection against homologous and heterologous H5N1 challenge (Leonard et al., 2025; Wang et al., 2025; Cheng et al., 2024). No major clinical or pathological effects of the vaccine have been found in safety testing conducted in vaccinated poultry and the platform appeared to be safe (Leonard et al., 2025; Wang et al., 2025; Snoeck et al., 2023).

In spite of such benefits, stability is a significant challenge for mRNA vaccines in poultry. The mRNA is sensitive to degradation which can result in significant demands for temperature control during storage and transport. This is especially difficult in large-scale vaccines in poultry production systems. Lyophilization and spray drying are therefore being explored to enhance RNA stability and prolong the shelf life of vaccines for storage at less stringent conditions (Chang et al., 2025).

The efficient delivery is also paramount. Currently, lipid nanoparticles (LNPs) are among the most promising mRNA delivery systems, due to their ability to encapsulate RNA and to confer protection from degradation, cell uptake and intracellular antigen expression. The immune responses obtained with LNP formulated mRNA vaccines have been mostly higher than those obtained with naked mRNA vaccines (Xu et al., 2023). Other delivery methods could further enhance their applicability in poultry. Another approach that has been explored is needle-free jet injection for the delivery of mRNA without the need for needles to penetrate the cell wall, which can still be efficiently taken up into cells (Chang et al., 2025). The mucosal route of administration (intranasal, aerosol, oral) is also of interest as it may generate local immunity at respiratory and other mucosal surfaces that are important sites of AIV entry (Supplementary Fig. 6) (Chang et al., 2025).

In general, mRNA vaccines have the features of a fast antigen redesign, high immunogenicity and a non-replicating vaccine platform. However, the use of these in poultry is still in an experimental phase. Sustained efforts to increase thermal stability, delivery efficiency, formulation cost and administration at a large scale will be necessary in order to make mRNA vaccines a viable alternative to existing AIV vaccination platforms.

### 4.4 Virus-like particles (VLPs)

VLPs are a promising platform for vaccine development for AIVs (Supplementary Fig.7). VLPs consist of viral structural proteins which self-assemble into the nanoscale, similar to native virions, but without any viral genetic material, and thus non-infectious. This structural mimicry retains the essential antigenic determinants and does not include the risk of replication as with live vaccines (Pushko et al., 2005; Kang et al., 2009; Schneider-Ohrum & Ross, 2011; Zhang et al., 2023). VLP antigenically relevant surfaces can be formed by structural proteins like HA, NA and matrix proteins, especially M1, which can drive VLP formation in influenza (Kang et al., 2009; Zhang et al., 2023; Yanase et al., 2013). The repetitive structure of viral antigens enables efficient immune recognition, B-cell activation, and production of neutralizing antibodies (Pushko et al., 2007; Chang et al., 2011; Mallick et al., 2011; Liu et al., 2016) and promotes cellular immune responses through the MHC-I and MHC-II antigen presentation pathways.

Several expression systems have been developed to produce AIV VLPs, such as baculovirus vectors in insect cells, mammalian cells and platforms in plant cells, which offers flexibility in the production and scale-up of vaccines (Zhang et al., 2023; Yanase et al., 2013). They have a proven protection efficacy against a number of AIV subtype(s).

VLP vaccines derived from H9N2 have shown to be 100% protective against lethal viral challenge in mice and ferrets, and have shown significant reduction in morbidity and mortality (Pushko et al., 2005; Pushko et al., 2007). Use of immunostimulatory adjuvants, like CpG-ODN, can also improve the antibody response and protective efficacy (Mallick et al., 2011). VLP-Gag derived from mouse leukemia virus (MLV) have also been found to elicit higher hemagglutination inhibition titers and greater protection in comparative studies compared to VLPs based on influenza M1 (VLP-M1).

One of the major advantages of VLPs is the potential to alter the particle composition and the antigenic display. Molecular adjuvants such as flagellin (FliC) or granulocyte-macrophage colony-stimulating factor (GM-CSF) could be included to enhance immune activation and antibody responses (Liu et al., 2016). Alternatively, VLPs can be engineered to contain antigens from different influenza subtypes, which may be useful for the development of multivalent vaccines. For example, neutralization antibody activity of H5H7 and H1H5H7 VLP was studied against different hemagglutinin subtypes which would offer a strategy to achieve cross protection against antigenically distant AIV strains (Schneider-Ohrum & Ross, 2011; Hong et al., 2019).

VLPs are generally safe and flexible vaccine platforms with structural features required for high immune recognition. They have been designed as broad and potentially cross-protective AIV vaccines as they can present influenza antigens in a virus-like conformation, tolerate multiple AIV antigens and contain immune-stimulatory components (Pushko et al., 2005; Kang et al., 2009; Schneider-Ohrum & Ross, 2011; Zhang et al., 2023; Yanase et al., 2013; Pushko et al., 2007; Chang et al., 2011; Mallick et al., 2011; Liu et al., 2016; Hong et al., 2019).

### 4.5 Vaccine failures and challenges in avian influenza control

Several interacting factors, such as antigenic variation, incomplete vaccination coverage, maternal antibody interference, and viral persistence in reservoir hosts, are responsible for the failure of avian influenza vaccines. Vaccination should therefore be combined with surveillance, biosecurity and vaccination strategies tailored to the local epidemiological conditions. Studied showed that these factors include: i) **Antigenic variation;** In Indonesia and Egypt, vaccine failures have been associated with the emergence of new H5N1 variants that were not well matched to licensed vaccines, leading to continued virus circulation despite vaccination campaigns (Kim et al., 2010; Swayne et al., 2015; Kayali et al., 2016). ii) **Maternal immunity;** In Egypt, it was reported that maternally derived antibodies from vaccinated hens interfere with the efficacy of vaccination in chicks, highlighting the importance of considering maternal antibody titers in the design of vaccination schedules (Abdelwhab et al., 2012). iii) **Reservoir Hosts, Limited Coverage;** Vaccination of ducks is limited because ducks are capable of carrying and shedding the virus with mild or no clinical disease, which is a prerequisite for viral persistence and transmission. Inadequate vaccination coverage remains a major barrier to effective disease control (Zhao et al., 2024). Iv) **Viruses evolution;** Vaccination can exert selective pressure on the viruses circulating in a population, which favors the selection or persistence of antigenic variants with reduced susceptibility to vaccine-induced immunity. Therefore, ongoing antigenic surveillance and timely vaccine updating are essential to sustain vaccine effectiveness (Wang et al., 2012). (Supplementary Table 8). Collectively, these factors emphasize the need for antigenically appropriate vaccines, adequate population coverage and appropriate timing of vaccination, surveillance and effective biosecurity measures for successful avian influenza control.

Section summary. The next-generation platforms discussed in this section are expected to provide solutions to many of the drawbacks of traditional avian influenza vaccination. Experimental studies have reported high levels of protection for recombinant subunit, viral-vector, mRNA and VLP-based vaccines. However, the magnitude and breadth of protection differ according to the antigen, host species, formulation, vaccination schedule, challenge strain and experimental endpoint. mRNA and recombinant antigen technologies have the advantage of design flexibility and the potential for rapid updates, whereas VLPs and selected viral vectors can improve antigen presentation and immunogenicity. However, due to differences between experimental models and unavailability of standardized field data, direct comparisons of the reported protection percentages between different platforms are not possible.

## 5 Broadly-neutralizing antibodies (bNAbs)

While next-generation platforms provide improved flexibility and delivery options, achieving broader and more durable protection also requires strategies targeting conserved viral features and optimizing the quality and breadth of host immune responses.

The production of vaccines that elicit bNAbs is a critical approach to broad and more lasting immunity to AIVs. Conventional vaccines based on strain-specific epitopes may not be as effective because of continuous antigenic variation of the AIVs. Current strategies emphasize conserved epitopes, especially in HA, as well as vaccination strategies and platforms which are capable of redirecting and expanding the antibody response (Fig. 2).

The conserved HA stem is a key target for the development of bNAb vaccines. The stem region is structurally more conserved across various influenza strains and is less immunodominant than the HA head, which is highly variable, and is thus an attractive target for broadly protective immunity (Wei et al., 2012).

Headless HA constructs have been used to develop immunogens that elicit stronger responses to conserved HA stem epitopes and the generation of antibodies with more cross-reactive binding activity (Gromadzka et al., 2022). Further extension of antibody responses can be achieved by sequential vaccination of antigenically different influenza strains or heterologous prime-boost regimes. For instance, a priming/boosting approach using inactivated or viral-vector vaccines has been studied to increase cross-reactive immunity and thus increase protection against a wide variety of AIV strains (Wei et al., 2012).

There are several platforms tested for the potential to induce broad humoral and mucosal immunity. Attenuated viral and bacterial vectors, such as *Salmonella Gallinarum*, can be used to induce mucosal IgA, systemic IgY and CD8⁺ T-cell responses, providing several layers of protection against AIV infection (Zhao et al., 2022). Viral shedding has also been reduced and mucosal immune responses have been boosted using virosome-based vaccines alone, or in combination with immunostimulatory agents, like CpG-ODN or recombinant interferon-γ (Mallick et al., 2012). Likewise, influenza antigens can be presented by VLP-based vaccine in a very repetitive and virus-like presentation. To date, however, industrially produced H5N1 HA-NA-M1 VLPs have been shown to induce strong anti-HA stem antibodies in hens, which is a testament to the potential of VLPs for broad protection against AIV (Ellebedy et al., 2014).

These vaccines can also generate larger and longer lasting antibody responses with the help of adjuvants. In addition, use of squalane-based emulsions or other adjuvants can enhance antigen presentation and enhance the duration of immunologic stimulation, leading to more antibody production (Mallick et al., 2012; Parra-Rojas et al., 2019; Dollinger et al., 2025). The combinations of CpG motif with monophosphoryl lipid A (MPLA) and squalene-based formulations have resulted in enhanced humoral and cellular responses, such as higher antibody titers and better protection against AIV challenge (Dollinger et al., 2025). For instance, the formulations with the addition of ISA78VG and Quil-A have demonstrated full seroconversion and high titers of hemagglutination inhibition (Dollinger et al., 2025).

One of the biggest challenges to induction of bNAbs is the immunodominance of variable HA epitopes. Immunity targeting the HA head may hone in on unique epitopes and therefore contribute to a narrow spectrum of protection (Ellebedy et al., 2014; Gromadzka et al., 2022). Design of rational immunogens is thus needed to redirect B-cell responses to the conserved HA-epitopes. Computational methods and structure-based vaccine design may help identify conserved neutralizing epitopes, as well as optimizing the structures of antigens for increased antibody-antigen interaction (Lin et al., 2018; Gromadzka et al., 2022; Peng et al., 2014). These methods have special significance in tackling antigenic drift and shift of HA and NA, which is continuous and may affect the effectiveness of the strain-specific immunity (Lin et al., 2018; Gromadzka et al., 2022; Peng et al., 2014).

AIVs also have mechanisms which can inhibit the development of effective antiviral immunity. NS1 protein is a prominent inhibitor of type I interferon signaling with the ability to inhibit early innate antiviral defenses (Zhu et al., 2013). Other viral proteins such as PB1-F2 and the viral polymerase complex are able to interfere with interferon production and signaling (Zhu et al., 2013). AIV infection can also occur to affect the function of dendritic cells and antigen presentation, which may in turn reduce subsequent adaptive immune responses (Yang et al., 2016). The mechanisms demonstrate the need for vaccine strategies that elicit broad neutralizing antibodies and strong cellular immunity.

Conserved-antigen targeting and optimized adjuvants, delivery systems, and antigen-display technologies are thus being combined in next-generation AIV vaccines. mRNA and DNA vaccines are flexible vaccine platforms to express conserved influenza antigens and can induce both humoral and cellular immune responses (Xu et al., 2023; Xu et al., 2023; Snoeck et al., 2026).

Flu vaccines made with nanoparticles that present influenza antigens in repetitive arrays have also evoked robust HI Abs and neutralized Abs and shown protection against lethal AIV challenge (Chen et al., 2025). Oral recombinant vaccines using baculovirus or yeast system have been demonstrated to induce both mucosal and systemic immunity and have proven effective against homologous and heterologous AIV strains (Dollinger et al., 2025). In addition, vaccines that induce the display of conserved influenza epitopes on modular capsomeres and dendritic-cell targeting vaccines that enhance antigen presentation have shown promising results in pre-clinical studies (Lin et al., 2018; Gromadzka et al., 2022; Peng et al., 2014).

In sum, the induction of bNAbs to conserved AIV epitopes is an important aspect of the next generation influenza vaccine development. A combination of structure-guided antigen design, immunodominance management, right adjuvants and advanced vaccine platforms could enhance the general and long-lasting protection against antigenically diverse AIV strains. However, further research into antigenic evolution, immune evasion and host immune responses to AIV is still required to develop broadly protective and potentially universal vaccines against AIV (Chen et al., 2025).

*Section summary:* Besides the development of better vaccine delivery systems, strategies are needed to overcome the immunological limitations of strain-specific responses for broad and durable protection. Strategies such as targeting conserved influenza antigens or epitopes, optimizing antigen presentation, improving mucosal immunity, and rational design of immunogens to redirect immune responses to conserved regions may help to achieve broader protection. Of note, strategies to induce bNAbs have the potential to reduce the need for antigenic matching. However, further testing with antigenically diverse and contemporary avian influenza viruses will be required to determine the breadth, durability, and practical relevance of these approaches (supplementary Figure 8).

## 6 Conclusion: vaccine platforms & future directions

The geographical mismatch between capacity to develop advanced AIV vaccines and disease burden is one of the major hurdles to global implementation. Although H5N1 and H9N2 are still a threat to poultry production in areas where they are endemic, most of the research, development and manufacturing of next generation vaccines is currently focused mainly in North America and Europe. Laboratory estimates of world-level influenza vaccine manufacturing capacity suggest that the bulk of the capacity resides within a few manufacturers in high-income countries with only a small proportion in low- and middle-income countries where H5N1 and H9N2 are endemic (Simancas-Racines et al., 2025). A similar pattern has been described for veterinary AIV vaccines: bibliometric analysis of AIV vaccine-related research publications and licensed vaccine manufacture between 2019 and 2023 showed that the output of publications and registered vaccines was not proportionately related to the disease burden in Domínguez-Odio et al. (2025). North America and Europe, but to North American and European institutions and manufacturers (Domínguez-Odio et al., 2025). This split restricts technology transfer, local testing to prove the technologies, and equitable access to new and advanced vaccination technologies, and is one of the structural hurdles next generation platforms must overcome to sit alongside, and not alongside, traditional vaccines in the AIV affected regions.

To summarize, an integrated approach of optimized vaccination strategies together with genomic surveillance, biosecurity measures and rapid response to outbreaks will be needed for effective avian influenza prevention. Elucidation of antigenic variation of influenza viruses, coupled with further developments in immunogen design and engineering, could also be the foundation for future vaccines that could be more broadly protective and improve influenza preparedness worldwide against new threats.

## Using artificial intelligence bots

we utilized AI-powered tools to check grammar and enhance the academic quality of the text, which was primarily written by the authors.

In preparation of schematic figures Artificial intelligence (AI) was used to improve the visual presentation and facilitate the understanding of the scientific content. All scientific concepts, information, labels and textual content present in the figures were created by the authors based on the relevant scientific literature. AI was used only as a visualization and an illustration tool, under detailed instructions of the authors. The AI has not created or altered any scientific information, interpretation or conclusions. The figures have been checked for scientific accuracy and consistency with the cited literature. The schematic figures in full size are shown in the Supplementary figures.

## Statement for study

This article is a review study and does not include any new experiments involving animals performed by the authors, ethical approval and informed consent for animal use were not required. All information discussed in this review was obtained from previously published studies.

## Ethics approval and consent to participate

Not applicable.

## Consent for publication

Not applicable.

## Funding

No specific funding was received for this work.

## CRediT authorship contribution statement

**Mahboobeh Nazari:** Writing – review & editing, Writing – original draft, Project administration, Investigation, Conceptualization. **Mohammad Mahdi Mehrazar:** Writing – original draft.

## Declaration of competing interest

The authors declare that they have no known competing financial interests or personal relationships that could have appeared to influence the work reported in this paper.

## Data Availability

Not applicable. This article is a review and does not report original datasets.
