# Supplementary material for: Advances and challenges in avian influenza vaccines: From conventional approaches to next-generation strategies
Source: Vet Anim Sci. 2026 Sep 27;34:100883. doi: 10.1016/j.vas.2026.100883 (PMC13652967; doi:10.1016/j.vas.2026.100883)
Supplement: Supplementary file 1 [file mmc1.docx]

**Supplementary Tables**

**Supplementary Table 1: Summary of Historical H5 HPAI Outbreaks**

| Year | Event | Clade/Lineage | Importance |
| --- | --- | --- | --- |
| 1996 | Goose/Guangdong strain identified in China | Goose/Guangdong lineage | Ancestor of all modern H5 HPAI viruses |
| 1997 | Hong Kong poultry and human outbreak | Early H5N1 | First documented human H5N1 infections |
| 2003-2004 | Expansion throughout East and Southeast Asia | Multiple H5N1 clades | Beginning of widespread regional epidemics |
| 2005-2006 | Spread into Europe, Middle East, and Africa | Clade 2.2 | First large intercontinental spread by migratory birds |
| 2006 | Nigeria outbreak | Clade 2.2 | First confirmed H5N1 outbreak in Africa |
| 2014-2015 | Emergence of H5Nx reassortants (H5N8, H5N2) | Clade 2.3.4.4 | Demonstrated extensive reassortment and global dissemination |
| 2020-present | Global panzootic affecting poultry, wild birds, and mammals | Clade 2.3.4.4b | Largest HPAI epidemic recorded worldwide; major impact on poultry production and wildlife |
| Recent years | Continued circulation in Asia | Clade 2.3.2.1c | Persistent regional lineage with implications for vaccine matching |

**Supplementary Table 2: Host-Based Classification of** **Influenza Viruses**

| Host category | Representative influenza subtypes | Biological significance |
| --- | --- | --- |
| Wild aquatic birds (natural reservoir) | H5N1, H5N8, H7Nx, H9N2 and numerous other H1–H16/N1–N9 combinations | Primary natural reservoir; maintenance of influenza A diversity; long-distance dissemination through migration |
| Domestic poultry | H5N1, H5N8, H7N9, H9N2 | Major amplification hosts; responsible for economic losses and source of zoonotic transmission |
| Humans | H5N1, H5N8 (rare), H7N9, H9N2 (sporadic) | Zoonotic infections with varying clinical severity; pandemic concern |
| Mammals (spillover hosts) | H5N1 predominantly | Foxes, minks, cats, dogs, sea lions, seals and other mammals infected through spillover; evidence of expanding host range |
| Swine | Mainly H9N2-derived reassortants and occasional avian influenza viruses | Important "mixing vessel" facilitating reassortment between avian and mammalian influenza viruses |

Supplementary Table 3: comparison of avian protein structure and vaccine potential

| Antigen | Function | Structure | Antigenic Variation | Vaccine Potential |
| --- | --- | --- | --- | --- |
| HA | Receptor binding, membrane fusion | Trimeric glycoprotein, HA1 (head), HA2 (stem) | Extensive antigenic drift, 16 subtypes | Target for broad-spectrum vaccines, therapeutic antibodies |
| NA | Sialic acid cleavage, virus release | Surface glycoprotein, enzymatic activity | Less immunodominant, 9 subtypes | Potential vaccine antigen, high-quality antibodies |
| M2 | Ion channel activity, replication | Structural protein | Limited information | Not specified |
| NP | RNA replication and transcription | Internal protein | Limited information | Not specified |

**Supplementary Table 4: Comparative Summary of different types of inactivated vaccines**

| Aspect | Egg-Based Production | Cell-Culture-Based Production |
| --- | --- | --- |
| Time to Production | ~6 months | Faster |
| Yield Consistency | Variable | More consistent |
| Scalability | Limited | High |
| Contamination Risk | Higher | Lower |
| Pandemic Resilience | Vulnerable to egg supply issues | More resilient |

**Supplementary Table 5: Comparison of Different Adjuvants and Effectiveness**

| Adjuvant | Effectiveness |
| --- | --- |
| Aluminum Hydroxide | Induces humoral immune responses, effective protection in mice |
| Chitosan | Enhances hemagglutination-inhibiting and neutralizing antibodies, effective in mice and chickens |
| MF59 and AS03 | Enhances immune responses, effective in vulnerable populations |
| Mineral Oil-Based | Induces high antibody titers, effective in poultry |
| Synthetic Peptides | Enhances antibody responses, facilitates humoral and cellular immune responses |
| PolyI and AddaVax | Higher serum antibodies and IgG titers, promotes adaptive immune response |
| Naringenin | Increases IgG levels and hemagglutination inhibition titers, enhances immune responses |
| Lovastatin | Enhanced immunogenicity, increased antibody titers and cell-mediated responses |
| β-Glucan | Higher immune responses, increased hemagglutination inhibition titers |

**Supplementary Table 6: Summary of Key Strategies for Live Attenuated Vaccines**

| Strategy | Mechanism | Examples | Efficacy |
| --- | --- | --- | --- |
| Temperature Sensitivity | Mutations that restrict replication at higher temperatures | PA (F35S) mutation in H5N1, Cold-adapted H2N2 | Effective in reducing virulence and providing protection |
| Gene Deletion | Removal or truncation of specific viral genes | Truncated NS1 proteins | High efficacy in reducing mortality and morbidity |
| Combination | Integrating multiple attenuation strategies | QIV with TS mutations and epitope tags, miRNA target sites | Enhanced safety and cross-protection |

**Supplementary Table 7. comparison of mRNA vaccine with traditional avian influenza vaccines.**

| Aspect | mRNA Vaccines | Traditional Vaccines |
| --- | --- | --- |
| Mechanism | Synthetic mRNA encoding antigens | Inactivated or live-attenuated viruses |
| Development Speed | Rapid and scalable | Slow and labor-intensive |
| Immunogenicity | Strong, broad, and long-lasting | Limited, strain-specific |
| Cold-chain Logistics | Required | Less stringent |
| Public Acceptance | Potential hesitancy | Well-established |
| Antigenic Drift | Less affected, broad protection | Highly affected, frequent updates needed |
| Production Ease | Complex, requires advanced technology | Relatively easy |

**Supplementary Table 8: Key Factors Contributing to Vaccine Failures**

| **Factor** | **Description** |
| --- | --- |
| **Emergence of Variant Field Strains** | New virus variants can escape immunity provided by existing vaccines, leading to outbreaks. |
| **Mismatch Between Vaccine and Field Strains** | Vaccines may not be updated to match circulating strains, reducing their effectiveness. |
| **Maternal Antibody Interference** | Maternal antibodies in chicks can interfere with vaccine-induced immunity, causing failures. |
| **Incomplete Vaccination Coverage** | Unvaccinated populations (e.g., ducks) act as reservoirs, undermining control efforts. |
| **Logistical and Regulatory Challenges** | Issues with vaccine deployment, administration, and regulation can limit effectiveness. |
| **Evolutionary Pressure and Resistance** | Mass vaccination may drive the evolution of vaccine-resistant virus strains. |
| **Biosecurity and Control Measures** | Vaccination alone is insufficient without strong biosecurity and surveillance. |
