# Supplementary material for: Advances and challenges in avian influenza vaccines: From conventional approaches to next-generation strategies
Source: Vet Anim Sci. 2026 Sep 27;34:100883. doi: 10.1016/j.vas.2026.100883 (PMC13652967; doi:10.1016/j.vas.2026.100883)
Supplement: Supplementary file 2 [file mmc2.docx]

**Supplementary Figures**

**Supplementary Figure 1. Vaccination and its role in control and eradication of avian influenza. T**he figure shows the importance of vaccination as an integral part of integrated AIV control programs. Vaccination reduces clinical disease, mortality, viral replication and shedding and results in reduced virus circulation and transmission in poultry populations. Depending on the epidemiological situation, different vaccination strategies can be implemented (routine vaccination in endemic areas, emergency vaccination in outbreaks, preventive vaccination in high-risk areas).

**Supplementary Figure 2.** **Circulation, genetic diversity and evolution of H9 and H7 avian influenza viruses.** The figure summarizes the emergence, phylogenetic diversification and geographic spread of major H9 and H7 avian influenza lineages. The H9N2 lineage originated from the first isolated A/turkey/Wisconsin/1/1966 and then diverged into the North American and Eurasian lineages which comprise the predominant G1-like, Y280-like and Korean-like groups. The G1-like lineage has been spread across Asia, Central Asia and the Middle East whereas Y280-like viruses have been mostly circulated in China and Southeast Asia. H9N2 viruses continue to be of great relevance due to their ongoing evolution, their potential to reassort with other influenza subtypes and their contribution of internal genes to zoonotic influenza strains.

H7 influenza lineage has low and highly pathogenic variants. Major subtypes are H7N3, H7N9, H7N1 and H7N7. Most H7 viruses are low pathogenic in birds, but some variants, including H7N9 and H7N1/H7N7 strains, have shown zoonotic potential and have caused large outbreaks in poultry and humans. The figure highlights the role of wild birds and domestic poultry as reservoirs, the geographical distribution of the major lineages and the importance of genetic reassortment, antigenic evolution, surveillance and vaccination strategies in controlling H9 and H7 avian influenza viruses.

**Supplementary Figure 3. Structure of avian influenza virus and major antigenic targets involved in neutralizing immunity.** Diagram depicting the structure of AIVs and functions of major viral proteins involved in infection, immune recognition and vaccine development. HA and NA are the major surface glycoproteins and major targets of neutralizing antibodies. HA is a trimeric glycoprotein consisting of HA1 and HA2 subunits; the receptor-binding site (RBS) is located on the globular head of HA1, which mediates attachment of the virus to host cell sialic acid receptors, and the stalk region of HA2 mediates membrane fusion and is a major target of broadly neutralizing antibodies. Differences in receptor specificity for avian (α2,3-linked) and mammalian (α2,6-linked) sialic acid recognition are known to be involved in host adaptation and zoonotic potential. The figure shows major HA antigenic domains with important residues in the receptor-binding and antigenic sites of H5N1, where amino acid substitutions may lead to reduced antibody recognition and immune escape. In contrast, low antigenic diversity in the conserved HA stem region offers an attractive target for universal influenza vaccine strategies. Neuraminidase (NA) is a tetrameric receptor destroying enzyme which cleaves sialic acids on host cells and viral particles and facilitates release of progeny virions. NA-specific antibodies are less immunodominant than HA, but can inhibit viral release and thus represent an important complimentary target for vaccine and antiviral development. In addition to the viral proteins described above, other viral components are being considered as potential targets for next generation vaccine platforms, including the M2 ion channel, involved in virus uncoating, and the nucleoprotein (NP), which is required for viral RNA replication and transcription.

**Supplementary Figure 4. Strategies of attenuation and safety considerations of live attenuated avian influenza vaccines (LAIVs).** Schematic representation of the major genetic approaches for LAIV development including temperature-sensitive (TS) mutations, targeted gene deletions and multi-layered attenuation strategies. TS mutations in polymerase and nucleoprotein genes limit viral replication at elevated temperatures but allow replication at permissive temperatures. NS1 deletion/truncation and HA multi-basic cleavage site (MBS) deletion reduce pathogenicity by increasing innate immune activation and limiting viral spread. The 6:2 reassortant approach uses internal genes from a cold-adapted master donor virus, and HA and NA genes from circulating avian influenza strains to maintain attenuation and to provide relevant antigenic characteristics. Other approaches such as microRNA response element (MRE)-mediated attenuation and changes in polymerase fidelity can improve genetic stability and minimize the risk of reversion. LAIVs induce a broad range of immune responses including mucosal IgA, systemic antibody and cellular immunity which provide cross-protection against heterologous avian influenza viruses. Reversion to virulence and reassortment with wild-type viruses are major safety concerns that are managed by rational genetic design, reassortment-incompetent vaccine platforms, chimeric constructs, and controlled delivery strategies, such as *in ovo* vaccination.

**Supplementary Figure 5. Mechanism of action and major platforms of viral vector-based vaccines against avian influenza.** Recombinant viral vectors such as adenovirus, herpesvirus of turkey (HVT) and fowlpox virus are used to transfect the influenza HA gene into host cells, which results in the expression of HA, its presentation to the immune system and induction of humoral, cellular and mucosal immune responses. Also shown is the recombinant fowlpox based TROVAC-H5 vaccine and the αGal HyperAcute™ technology, which involves the addition of αGal epitopes to recombinant influenza antigens to improve immune recognition. All of these can decrease clinical disease, virus replication, and virus shedding after AIV challenge. Antigenic matching, vector immunity, vaccine dose, administration route, and the efficiency of antigen expression are key factors that impact vaccine performance.

**Supplementary Figure 6.** **Design, delivery routes, and mechanism of action of avian mRNA vaccines.** The schematic illustrates the major steps involved in the development and application of mRNA vaccines for poultry. (1) Vaccine design: selection of an appropriate pathogen-derived antigen is followed by optimization of the mRNA construct, including the 5′ cap, untranslated regions (UTRs), antigen-encoding open reading frame (ORF), and poly(A) tail. Modified nucleosides and avian-optimized codon usage can improve mRNA stability and reduce undesirable innate immune sensing. The mRNA is encapsulated within lipid nanoparticles (LNPs) composed of ionizable lipids, phospholipids, cholesterol, and PEG-lipids to facilitate protection, cellular delivery, and cytoplasmic release. (2) Routes of delivery: depending on the vaccine formulation and target pathogen, mRNA vaccines may be administered through in ovo, intramuscular, subcutaneous, ocular/nasal, spray, or oral routes, providing options for individual or mass vaccination of poultry. (3) Mechanism of action: following administration, LNP–mRNA particles are taken up by host cells through endocytosis. Endosomal escape releases the mRNA into the cytoplasm, where it is translated by ribosomes to produce the target antigen. The antigen is subsequently processed and presented through major histocompatibility complex (MHC) class I and class II pathways, activating CD8⁺ and CD4⁺ T cells and promoting B-cell activation and antibody production. Concurrent innate immune signaling, including type I interferon and cytokine responses, contributes to immune priming. The coordinated humoral and cellular responses generate protective immunity, potentially reducing pathogen replication, disease severity, and transmission in vaccinated birds.

**Supplementary Figure 7. Design, production, immunogenicity, and protective mechanism of virus-like particle (VLP) vaccines against AIV.** The schematic illustrates the development and immunological function of AIV VLP vaccines. (1) Design and production: AIV structural proteins, primarily hemagglutinin (HA), neuraminidase (NA), and matrix protein 1 (M1), are expressed using recombinant expression systems such as insect, mammalian, or plant cells. These proteins undergo self-assembly to generate VLPs that mimic the structural and antigenic characteristics of AIV virions but lack a viral genome and are therefore non-replicating and non-infectious. VLPs can be formulated with appropriate adjuvants to enhance immunogenicity. (2) Immunogenicity and mechanism of action: following administration, VLPs are taken up by antigen-presenting cells (APCs), where the constituent antigens are processed and presented through MHC class I and class II pathways. This promotes CD8⁺ cytotoxic T-cell and CD4⁺ helper T-cell responses, respectively, while also activating B cells and plasma cells to produce AIV-specific neutralizing antibodies. VLP uptake may also stimulate innate immune responses, including type I interferon and cytokine production, and promote dendritic-cell maturation. (3) Protective immunity: vaccination induces coordinated humoral and cellular immune responses that can limit viral replication and reduce clinical signs, morbidity, mortality, and viral shedding following exposure to virulent AIV. The modular nature of VLPs also enables incorporation of antigens from different AIV subtypes, supporting the development of multivalent formulations targeting strains such as H5, H7, and H9N2.

**Supplementary Figure 8. Evolution of avian influenza vaccination strategies from conventional to broadly protective approaches.** Conceptual evolution of avian influenza vaccine development from conventional strain matched approaches toward universal vaccine strategies Traditional inactivated and live attenuated vaccines have offered effective control of diseases, but they have limitations due to antigenic mismatch, incomplete mucosal protection, and difficulties in updating the vaccine. These limitations prompted the development of next-generation platforms that include viral-vector, recombinant subunit, DNA, mRNA and VLP vaccines that enable improved flexibility and broader immune activation. Future universal vaccine strategies will need to overcome viral diversity by targeting conserved antigens, structure-guided immunogen design, induction of broadly neutralizing antibodies and durable cross-protective immunity.

**Supplementary Fig. 1**

**Supplementary Fig. 2**

**Supplementary Fig. 3**

**Supplementary Fig. 4**

**Supplementary Fig. 5**

**Supplementary Fig. 6**

**Supplementary Fig. 7**

**Supplementary Fig. 8**
